# From eggs to adulthood: sustained effects of early developmental temperature and corticosterone exposure on physiology and body size in an Australian lizard

**DOI:** 10.1242/jeb.249234

**Published:** 2024-12-12

**Authors:** Ondi L. Crino, Kristoffer H. Wild, Christopher R. Friesen, Dalton Leibold, Naomi Laven, Amelia Y. Peardon, Pablo Recio, Karine Salin, Daniel W. A. Noble

**Affiliations:** ^1^ College of Science and Engineering, Flinders University, Bedford Park, SA 5001, Australia; ^2^ Division of Ecology and Evolution, Research School of Biology, The Australian National University, Canberra, ACT 2601, Australia; ^3^ School of BioSciences, The University of Melbourne, Parkville, VIC 3010, Australia; ^4^ School of Earth, Atmospheric and Life Sciences, University of Wollongong, Wollongong, NSW 2522, Australia; ^5^ Ifremer, Laboratory of Environmental Marine Sciences, University Brest, CNRS, IRD, 29280 Plouzané, France

**Keywords:** Cellular metabolism, Glucocorticoids, Incubation, Mitochondria, Stress, Temperature

## Abstract

Developing animals are increasingly exposed to elevated temperatures as global temperatures rise as a result of climate change. Vertebrates can be affected by elevated temperatures during development directly, and indirectly through maternal effects (e.g. exposure to prenatal glucocorticoid hormones). Past studies have examined how elevated temperatures and glucocorticoid exposure during development independently affect vertebrates. However, exposure to elevated temperatures and prenatal corticosterone could have interactive effects on developing animals that affect physiology and life-history traits across life. We tested interactions between incubation temperature and prenatal corticosterone exposure in the delicate skink (*Lampropholis delicata*). We treated eggs with high or low doses of corticosterone and incubated eggs at 23°C (cool) or 28°C (warm). We measured the effects of these treatments on development time, body size and survival from hatching to adulthood and on adult hormone levels and mitochondrial respiration. We found no evidence for interactive effects of incubation temperature and prenatal corticosterone exposure on phenotype. However, incubation temperature and corticosterone treatment each independently decreased body size at hatching and these effects were sustained into the juvenile period and adulthood. Lizards exposed to low doses of corticosterone during development had elevated levels of baseline corticosterone as adults. Additionally, lizards incubated at cool temperatures had higher levels of baseline corticosterone and more efficient mitochondria as adults compared with lizards incubated at warm temperatures. Our results show that developmental conditions can have sustained effects on morphological and physiological traits in oviparous lizards but suggest that incubation temperature and prenatal corticosterone do not have interactive effects.

## Teaser

**Highlighted Article:** Incubation temperature and prenatal corticosterone exposure have independent sustained effects, but not interactive effects, on morphological and physiological traits in the delicate skink.

## INTRODUCTION

Climate change is one of the most ubiquitous anthropogenic disturbances currently experienced by wildlife. With global temperatures increasing at an unprecedented rate (Diffenbaugh and Field, 2013; Tingley and Huybers, 2013), there is an urgent need to understand the physiological capacity of organisms to respond to elevated temperatures, and how such responses affect individual fitness and population dynamics (Chown et al., 2010; Fuller et al., 2010; Helmuth et al., 2005). Developing animals may be particularly sensitive to elevated temperatures associated with climate change because they are generally less able to behaviourally regulate their body temperature compared with adults (but see Du and Shine, 2022). Additionally, developmental conditions can profoundly affect morphology, physiology and behaviour (Eyck et al., 2019; Monaghan, 2008; Nord and Giroud, 2020). Such developmental effects can affect fitness across life-history stages and can be transmitted across generations through intergenerational effects (e.g. Bath et al., 2018; Crino et al., 2014b; Kraft et al., 2021; Mitchell et al., 2013).

Developing animals can be affected by elevated temperatures directly through interactions with their environment, and indirectly through maternal effects. For example, in vertebrates, exposure to stressors or disturbances such as elevated temperature can increase maternal glucocorticoid hormone levels, which can, in turn, have sustained effects on developing animals (reviewed in Crino et al., 2024; Mentesana and Hau, 2022). The independent effects of elevated temperatures and glucocorticoids on developing animals have been well studied (reviewed in Crino and Breuner, 2015; Nesan and Vijayan, 2013; Noble et al., 2018; Seckl and Meaney, 2004; Weeks et al., 2022). However, few studies have tested the combined effects of elevated temperatures and glucocorticoids on developing animals despite the recognition that glucocorticoids are likely to play an important role in shaping individual and population responses to global climate change (Crino et al., 2024; Mentesana and Hau, 2022; Names et al., 2024; Sumasgutner et al., 2023; Taff et al., 2024).

The effects of elevated temperatures on developing animals have been studied extensively in relation to incubation temperature in oviparous reptiles (Booth, 2018; DuRant et al., 2013; Jonsson and Jonsson, 2014; Noble et al., 2018). Incubation temperature is known to affect a range of traits depending on the species, including sex, growth, behaviour, locomotor performance, metabolism and reproductive success (e.g. Braña and Ji, 2000; De Jong et al., 2023; Esquerré et al., 2014; Kar et al., 2022; Warner and Shine, 2008). For example, in oviparous reptiles, exposure to high temperatures during incubation can accelerate embryonic development, resulting in individuals that hatch quickly but at a smaller body size than individuals exposed to cooler incubation temperatures (Dayananda et al., 2017; Kar et al., 2024). Elevated incubation temperatures can also have sustained effects on mitochondrial respiration and metabolic enzymes, suggesting that developmental conditions can affect growth at later life-history stages through sustained changes in cellular metabolism (Seebacher and Grigaltchik, 2014; Sun et al., 2015).

Similar to incubation temperature, exposure to glucocorticoids during development can affect many aspects of physiology, behaviour and performance (reviewed in Crino and Breuner, 2015; Eyck et al., 2019; McGowan and Matthews, 2018; Nesan and Vijayan, 2013). Glucocorticoids are steroid hormones that play important roles in vertebrate metabolism and stress responses (McEwen and Wingfield, 2003; Picard et al., 2014; Wingfield and Kitaysky, 2002). Glucocorticoids promote physiological and behavioural responses that allow animals to cope with disturbances and are thus considered mediators of adaptive responses to environmental conditions (Sapolsky et al., 2000). Developing animals can be exposed to glucocorticoids from maternal sources (during gestation, *in ovo* and from breastmilk in mammals) and from their own endogenous production in response to postnatal disturbances (e.g. food restriction, environmental conditions, parental interactions; Crino and Breuner, 2015; Monaghan and Haussmann, 2015). Exposure to elevated glucocorticoids during development affects a range of phenotypic traits, including cellular metabolism, growth and development, body condition, immune function and reproductive strategies (Blas et al., 2007; Casagrande et al., 2020; Crino et al., 2014a,b; Grindstaff and Merrill, 2017; MacLeod et al., 2018). Additionally, exposure to glucocorticoids during development can have sustained effects on the neuroendocrine pathway that regulates the production of glucocorticoids (the hypothalamic–pituitary–adrenal or HPA axis), resulting in the secretion of higher levels of glucocorticoids later in life (e.g. Crino et al., 2022; Pakkala et al., 2016; Spencer et al., 2009). In this way, developmental conditions that change HPA axis function can indirectly affect traits at later life-history stages that are influenced by glucocorticoids (e.g. mitochondrial function and sexual trait expression; Crino et al., 2022).

Many studies have examined how elevated temperatures and glucocorticoid exposure during development independently affect vertebrates. However, their combined effects have not been rigorously tested despite the fact that they can be biologically linked. In ectotherms, elevated environmental temperatures have been associated with increased glucocorticoid levels in adults (Liu et al., 2020; Racic et al., 2020). Maternal glucocorticoids can be transmitted to developing offspring in both oviparous (Uller et al., 2009) and viviparous lizards (Itonaga et al., 2011) and affect phenotypic traits with possible consequences for fitness (De Fraipont et al., 2000; Vercken et al., 2007). Thus, during development, oviparous animals could be exposed to both elevated levels of glucocorticoids via maternal transmission and elevated temperatures during incubation. These developmental effects could be sustained across life-history stages if exposure to glucocorticoids during development changes HPA axis function resulting in elevated secretion of glucocorticoids across life. Glucocorticoids affect metabolism and thermal tolerance through interactions with thyroid hormones and mitochondria (Debonne et al., 2008; Picard et al., 2014). Sustained changes in glucocorticoid secretion could thus play important roles in regulating phenotypic responses to temperatures through changes in mitochondrial function. Multiple ‘stressors’ can have additive, synergistic or antagonistic effects on traits (Kaunisto et al., 2016; Padda and Stahlschmidt, 2022; Todgham and Stillman, 2013). Therefore, evaluating interactions between environmental factors (e.g. developmental temperature and maternal glucocorticoids) can be valuable for understanding the impact of complex environmental disturbances on animal life history and physiology (Padda and Stahlschmidt, 2022).

Here, we tested the long-term effects of prenatal exposure to elevated incubation temperature and corticosterone (the main glucocorticoid in lizards) on body size and growth, hormone responses, mitochondrial bioenergetics and survival in the delicate skink (*Lampropholis delicata*). We exposed lizards to one of two corticosterone treatments (high corticosterone, low corticosterone) or a control treatment *in ovo* to mimic elevated levels of maternal corticosterone. We then incubated eggs at either low (23°C) or high (28°C) incubation temperatures (representing the approximate range of incubation temperatures in natural nests; Cheetham et al., 2011). We measured body size and condition in response to developmental treatments at hatching and two additional time points over a ∼1.5 year period. After 1.5 years, we measured hormone levels (corticosterone, thyroxine and testosterone – males only) and mitochondrial bioenergetics from liver tissue in adults. We had four main predictions that related to growth and body size, adult endocrine function, adult mitochondrial function, and hatching success and survival. (1) Growth and body size – lizards treated with corticosterone prenatally would be smaller than control lizards at hatching and throughout life because of the sustained effects of corticosterone exposure during development on HPA axis function (see below). Lizards incubated at warmer temperatures would be smaller at hatching than lizards incubated at cool temperatures, but differences in body size would not be present later in life. Further, high incubation temperature would interact synergistically with corticosterone treatment such that lizards exposed to both these treatments would be smaller than lizards from all other treatments. (2) Adult endocrine function – lizards treated with corticosterone prenatally would have higher baseline corticosterone levels as adults compared with control lizards as a result of the programmatic effects of corticosterone exposure during development on HPA axis function (Crino and Breuner, 2015; Eyck et al., 2019; Schoech et al., 2011). Males with higher baseline corticosterone levels would have lower testosterone levels because of the suppressive effects of glucocorticoids on sex steroid synthesis (Wingfield and Sapolsky, 2003). Developmental corticosterone treatment would affect adult thyroxine levels because corticotropin releasing factor (a hormone associated with the HPA axis) can stimulate the neuroendocrine pathway that regulates thyroid hormone production (De Groef et al., 2006; Geris et al., 1996). Thyroxine levels would be positively associated with growth among individuals given the role of thyroid hormones in regulating growth and metabolism (Gerwien and Johnalder, 1992; McNabb, 2007). (3) Mitochondrial bioenergetics in liver tissue and growth – similar to past studies, developmental treatments would have sustained effects on adult mitochondrial respiration (Crino et al., 2022; Stier et al., 2022) such that lizards exposed to elevated corticosterone during development have less efficient mitochondria as adult Additionally, we predicted that lizards incubated at higher temperatures would exhibit changes in mitochondrial function that would enable them to meet the heightened metabolic demands imposed by elevated temperature (Seebacher and Grigaltchik, 2014; Sun et al., 2015). (4) Survival – lizards treated with corticosterone during development would have lower survival than control lizards, and high incubation temperatures and corticosterone treatment would interact to further decrease survival.

Our research builds on recent research that examines the sustained effects of prenatal exposure to high temperatures on whole-animal metabolic rate and growth (De Jong et al., 2023; Kar et al., 2024) by testing the joint effects of elevated temperatures and corticosterone treatments on hormone levels, mitochondrial bioenergetics, and phenotypic and survival outcomes. Additionally, our study tests physiological mechanisms (mitochondrial bioenergetics and corticosterone levels) that may link maternal and developmental effects to sustained responses to elevated temperatures.

## MATERIALS AND METHODS

### Lizard husbandry and housing

This study was conducted from November 2021 to June 2023 using a colony of delicate skinks at The Australian National University (Canberra, Australia). Delicate skinks, *Lampropholis delicata* (De Vis 1888), are native to eastern Australia, occupy various habitats, and are commonly found in human-altered and urban areas (Cooger, 2014; Wilson and Swan, 2013). Delicate skinks reach sexual maturity at 1 year of age, are oviparous, and have a life span of ∼2–4 years (Forsman and Shine, 1995; Greer, 1989; Heatwole and Taylor, 1987). They are easily housed and bred in captivity and are a highly tractable species for empirical studies that test the long-term effects of developmental conditions (e.g. De Jong et al., 2023; Kar et al., 2022).

Lizards were housed communally in terraria (width×length: 40×55 cm) in groups of 3–4 females with 2 males. Terraria contained non-stick mats as substrate, refuges (eucalyptus bark and half-cut PVC pipe), a water container and a container full of moist vermiculite for egg laying. Terraria were heated by heat chords to provide a thermal gradient (22–32°C) to allow lizards to behaviourally thermoregulate and had UV lamps for UVA/UVB exposure. Mean preferred temperature in *L. delicata* ranges from 26 to 31°C and does not depend on the temperature experienced during development (Anderson et al., 2023; Zhang et al., 2023). Lights were set to a photoperiod of 12 h:12 h light:dark. Lizards were provided with water daily, crickets (*Acheta domestica*) every second day, and a calcium and multivitamin supplement once a week. All methods for housing, husbandry and experimental protocols were approved by The Australian National University Animal Ethics Committee (A2021/56).

### Experimental timeline

**Fig. 1.**
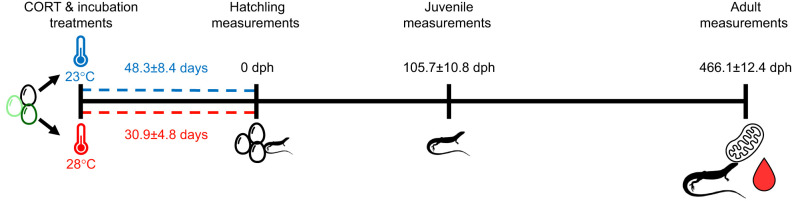
**Timeline of experimental protocol.** Eggs were dosed with one of three corticosterone (CORT) treatments (control, black; low corticosterone, light green; and high corticosterone, dark green) and then incubated at 23 or 28°C until hatching. We collected morphometric measurements [snout–vent length (SVL) and mass] following hatching and when lizards were juveniles (∼105.7 days post-hatching, dph) and adults (∼466.1 dph). We measured hormone levels [corticosterone, thyroxine and testosterone (males only)] and mitochondrial function from liver tissue in adult lizards.

Lizard enclosures were checked for eggs 3 days a week. Eggs were treated with hormone solutions the day they were found (∼24–72 h after they were laid; Fig. 1). Following treatments, eggs were incubated until hatching was recorded. Eggs were checked 3 days a week. On the day hatching was recorded, lizards were measured for snout–vent length (SVL) to the nearest millimetre using a ruler and body mass to the nearest milligram using a digital balance. After hatching, lizards were moved to solitary enclosures and provided with the same husbandry as lizards in the breeding colony (as above). We collected additional body size measurements when lizards were juveniles [mean±s.d. 105.7±10.8 days post-hatching (dph), range 85–123 dph] and when lizards were euthanised as adults at ∼1.5 years of age (466.1±12.4 dph, range 440–491 dph), at which point they were sexed by hemipene eversion. After euthanising lizards, we collected a blood sample for hormone analyses and liver tissue to measure mitochondrial bioenergetics. Body condition was calculated at each time point using the scaled mass index derived from SVL and body mass (Peig and Green, 2009). We calculated the growth rate from hatching to the juvenile period and hatching to adulthood as body size measurements as juveniles/adults minus body size at hatching divided by juvenile/adult age (dph).

### Experimental treatments

We exposed eggs to one of six corticosterone/temperature treatments in a fully factorial design. Eggs were assigned to treatment groups in a partially crossed split-clutch design such that eggs from a single clutch were randomly assigned across treatment groups. It was not possible to assign an egg from a single clutch to each of the treatment groups because mean clutch size is 3.0–4.4 eggs in *L. delicata* (Forsman and Shine, 1995). For hormone treatments, eggs were treated with either high corticosterone (10 pg mg^−1^) or low corticosterone (5 pg mg^−1^), or a control (vehicle) treatment. Corticosterone doses were selected based on published yolk corticosterone concentrations in other oviparous species (Hanover et al., 2019; Lovern and Adams, 2008), estimates of the percentage of steroids that are incorporated into the embryo following topical treatment (Crews et al., 1991; Vassallo et al., 2014), and preliminary measures of untreated eggs. Corticosterone treatments were made by dissolving crystalline corticosterone (Sigma, cat. no. C2505) in 100% ethanol. To dose eggs, we applied 5 µl of solutions to eggshells using a micropipette. Control eggs were treated with 5 µl of 100% ethanol. Following treatment with corticosterone or control solutions, eggs were incubated in covered plastic cups filled with damp vermiculite at either 23°C (hereafter cool) or 28°C (hereafter warm; representing the temperature extremes in natural nest sites in this species; Cheetham et al., 2011).

### Validation of corticosterone treatments

We measured corticosterone levels in a separate group of eggs to ensure that topical treatments increased corticosterone levels within a biologically relevant range. We dosed eggs with corticosterone treatments as above. We allowed eggs to incubate for 24±2 h at 28°C prior to removing the egg yolk. We used solid phase extraction (SPE) with silica-bonded vacuum columns (United Chemicals cat. no. CEC18156) to extract corticosterone from yolk samples and Arbor Assay Enzyme Immunoassay (EIA) kits (cat. no. K014) to measure corticosterone (full methods in Supplementary Materials and Methods). Corticosterone treatment increased mean yolk corticosterone levels 2.54 and 5.95 standard deviations above those of control eggs for low and high doses, respectively (see Results).

### Mitochondrial bioenergetics

Lizards were fasted for 72±4 h prior to euthanasia. Lizards were euthanised via an injection of Alfaxan (10 mg ml^−1^) followed by rapid decapitation. Immediately following decapitation, whole livers were removed, rinsed twice in 1 ml of ice-cold 1 mol l^−1^ phosphate buffered saline, and stored in 1 ml of ice-cold isolation buffer (250 mmol l^−1^ sucrose, 1 mmol l^−1^ EGTA, 20 mmol l^−1^ Tris HCl, pH 7.4 with KOH) prior to further processing (>30 min). We used differential centrifugation to isolate mitochondria (Lampl et al., 2015; Pallotti and Lenaz, 2001). Liver tissue was homogenised on ice with 3–4 gentle hand passes using a Potter–Elvehjem homogeniser. The homogenate was centrifuged at 4°C, 750 ***g*** for 10 min. The supernatant was transferred to a clean Eppendorf tube and centrifuged for a second time at 4°C, 750 ***g*** for 10 min. The supernatant was then transferred again to a clean Eppendorf tube and centrifuged at 4°C, 10,000 ***g*** for 10 min. The resulting supernatant was removed and the pellet containing isolated mitochondria was resuspended in 500 µl of MiR05 respiration media (0.5 mmol l^−1^ EGTA, 3 mmol l^−1^ MgCl_2_, 60 mmol l^−1^ potassium lactobionate, 20 mmol l^−1^ taurine, 10 mmol l^−1^ KH_2_PO_4_, 20 mmol l^−1^ Hepes, 110 mmol l^−1^ sucrose, 1 g l^−1^free fatty acid bovine albumin, pH 7.1 with KOH).

We measured mitochondrial oxygen consumption (pmol O_2_ s^−1^) using Oxygraph-2K high-resolution respirometers (Oroboros Instruments, Innsbruck, Austria) based on established methods (Brand et al., 1993; Doerrier et al., 2018; Salin et al., 2018) with minor modifications. Immediately following preparation, we added resuspended mitochondria to 1.5 ml of respiration media equilibrated at 30°C in one respiration chamber. We applied a series of mitochondrial substrates and inhibitors to measure oxygen consumption at five states: basal (state 2), maximal (state 3), leak (state 4) and residual oxygen consumption. Basal respiration was measured following the addition of pyruvate (5 mmol l^−1^), malate (2 mmol l^−1^) and succinate (10 mmol l^−1^), which support electron entry into the electron transport system via complexes I and II. Maximal respiration was induced with the addition of ADP (2 mmol l^−1^). Leak respiration was induced by adding oligomycin (2.5 µmol l^−1^), which inhibits ATP synthase. Oxygen consumption following the addition of oligomycin is attributed to proton leak across the inner mitochondrial membrane. Finally, we added antimycin A (2.5 µmol l^−1^), which inhibits mitochondrial complex III and allows for measurement of non-mitochondrial oxygen consumption. After administering antimycin A, we collected a 1 ml aliquot of the mitochondrial suspension from the respiration chamber to determine sample concentration. These samples were stored at −20°C until assayed using Coomassie Plus (Bradford) assays (Thermo Fisher Scientific, cat. no. 23236; Supplementary Materials and Methods).

Oxygen consumption values for basal, OXPHOS (state 3) and leak (state 4) respiration were corrected for non-mitochondrial oxygen consumption (following the addition of antimycin A) and protein content, yielding values in pmol O_2_ s^−1^ µg^−1^ of mitochondrial protein. We estimated mitochondrial efficiency as the respiratory control ratio (RCR), which is calculated as the ratio of oxygen consumed to drive the phosphorylation of ADP to ATP (OXPHOS) to oxygen consumed to offset proton leak across the inner mitochondrial membrane (Brand and Nicholls, 2011). A high RCR indicates that mitochondria have a high respiratory capacity for ATP production relative to the respiration required to offset proton leakage (Brand and Nicholls, 2011).

### Blood collection and plasma hormone analysis

We collected blood samples immediately after euthanising and decapitating lizards. Blood was collected from the trunk using heparinised microcapillary tubes within 6 min of disturbing lizards. The average time to collect blood samples was 2.4±0.8 min (range 1.5–5.8 min). In endothermic animals, glucocorticoid levels generally increase above baseline within 3 min of disturbance (Romero and Reed, 2005; Small et al., 2017). However, glucocorticoid levels in reptiles generally remain at baseline 5–15 min following disturbance (Cockrem, 2013; Tylan et al., 2020). We found no effect of the time to collect blood samples on plasma corticosterone levels (*P*=0.55, *F*_1,75_=0.36). Blood samples were kept on ice (<1 h) and then centrifuged at 7000 rpm for 7 min to separate plasma from red blood cells. The isolated plasma was frozen at −20°C prior to conducting hormone assays.

We measured corticosterone, thyroxine and testosterone (males only) from 5 µl of plasma. All hormones were measured from raw plasma diluted 1:100 using Arbor Assay EIA kits (cat. no. K014, K050, K032). All samples and standards were run in triplicate and all plates were read on a FLUOstar Omega microplate reader at 450 nm (full details in Supplementary Materials and Methods).

### Statistical analysis

Data were analysed in R version 4.4.0 using the lme4 (1.1.35.3), emmeans (1.10.2), performance (0.11.0) and car packages (3.1.2; http://R-Forge.R-project.org/projects/mumin/; https://CRAN.R-project.org/package=emmeans; Fox and Weisberg, 2019; Ludecke et al., 2021). We used a general linear mixed effects model (GLMM) with a Gaussian error distribution to test the effects of corticosterone treatments on yolk corticosterone levels. We also used GLMMs to test the effects of developmental treatments on incubation duration, body size and condition at hatching and across life, adult hormone levels, and adult mitochondrial bioenergetics. To control for lizards originating from the same clutch, we included clutch of origin as a random effect in all models. Initial models included an interaction term between temperature and corticosterone treatments. However, there were no interactions between temperature and corticosterone treatments for all analyses and the interaction term was removed from the final models (Table S1). We tested associations between growth and physiological parameters using GLMMs with growth of mass or SVL as dependent variables, thyroxine levels, corticosterone levels and mitochondrial respiration parameters as covariates, and sex as a fixed factor. We ensured that the underlying statistical assumptions of models were not violated by visually inspecting *Q–Q* plots, homogeneity of variance, variance inflation factors and model residuals using the ‘check_model’ function (Zuur et al., 2009). We tested differences between corticosterone treatments with pairwise comparison using ‘emmeans,’ corrected with the Tukey method. We conducted Pearson's Chi-squared test to determine the effects of developmental treatments on post-hatch survival across the duration of our study. Means are provided with one standard deviation unless indicated otherwise. Full model details and outputs can be found in Supplementary Materials and Methods.

## RESULTS

### Hormone treatment effects on yolk corticosterone levels

**Fig. 2.**
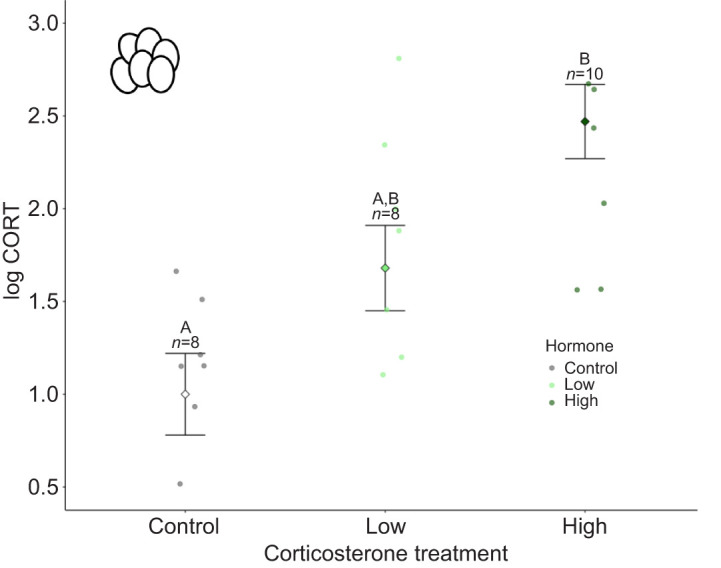
**Yolk corticosterone levels (log transformed) following control, low dose corticosterone and high dose corticosterone treatments.** Marginalised mean estimates (mean and s.e.) are provided based on a model that accounted for corticosterone developmental treatment and test plate effects on yolk corticosterone levels. Significant differences (*P*<0.05) from *post hoc* tests are indicated by different letters. Sample sizes (*n*) for each treatment are indicated above error bars.

Topical corticosterone treatment affected yolk corticosterone levels (*P*=0.002, *F*_2,22_=7.98; Fig. 2). Eggs treated with high doses of corticosterone had higher levels of yolk corticosterone than control eggs (*P*=0.002; high corticosterone 11.42±8.44 pg mg^−1^, control 3.09±1.40 pg mg^−1^, mean±1 s.d.). Eggs treated with low doses of corticosterone had yolk corticosterone levels intermediate between the high dose and control treatments (low corticosterone 6.64±4.92 pg mg^−1^). There were no differences in yolk corticosterone levels between eggs treated with high and low doses of corticosterone (*P*=0.11) and low doses of corticosterone and the control treatment (*P*=0.21).

### Developmental treatments across life – effects on incubation time, body size and condition

Lizards incubated at warm temperatures hatched faster than lizards incubated at cool temperatures (*P*<0.001, *F*_1,115_=1008.42; average days to hatch: warm 30.9±4.8 days and cool 48.3±8.4 days). There was no effect of corticosterone treatment on the time for lizards to hatch (*P*=0.52, *F*_2,115_=1.31; average days to hatch: high corticosterone 39.5±11.0 days, low corticosterone 40.6±11.5 days, control 39.8±11.0 days).

**Fig. 3.**
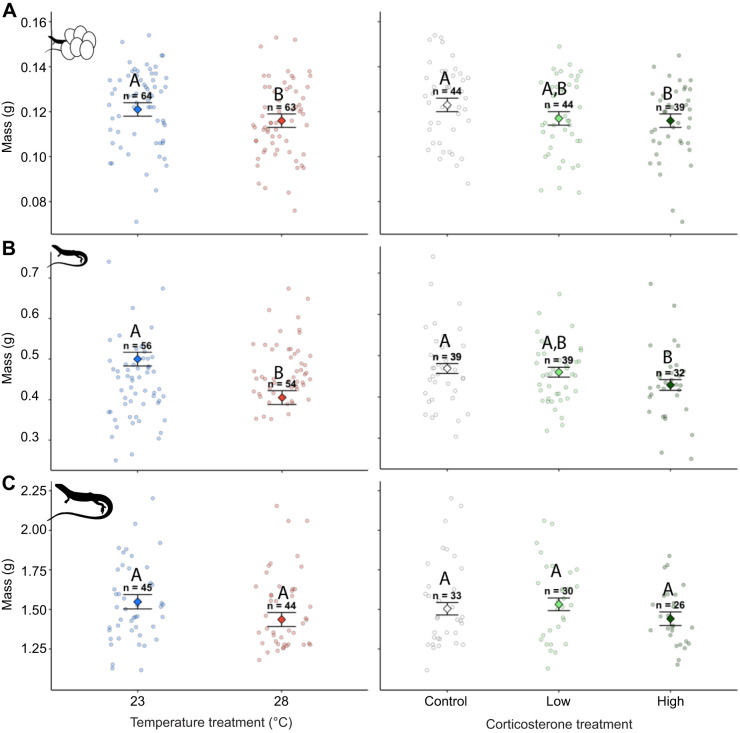
**Body mass in lizards exposed to incubation temperature (left) and prenatal corticosterone treatments (right).** Body mass is shown at hatching (A), in juveniles (B) and at adulthood (C). Significant differences (*P*<0.05) from main effects of incubation temperature and *post hoc* tests for differences between corticosterone treatments are indicated by different letters, and sample sizes (*n*) for each treatment are indicated above error bars. Marginalised mean estimates (mean and s.e.) are provided based on a model that accounted for incubation temperature, hormone treatment, body size, age and clutch ID as a random factor.

Incubation temperature did not affect SVL at hatching (*P*=0.99, *F*_1,123_=0.01; Fig. S1) but did affect body mass such that lizards incubated at warm temperatures weighed less than lizards incubated at cool temperatures (*P*=0.017, *F*_1,123_=5.71; Fig. 3A) and had lower body condition (*P*=0.03, *F*_1,123_=4.31). As juveniles, lizards incubated at warm temperatures during development had smaller SVL (*P*=0.01, *F*_1,101_=6.42) and weighed less compared with lizards incubated at cooler temperatures (*P*<0.001, *F*_1,101_=10.73; Fig. 3B), but there were no differences in body condition (*P*=0.91, *F*_1,101_=0.01). The effects of incubation temperature on body size that we observed at early ages were not present in adults. Incubation temperature did not affect adult SVL (*P*=0.35, *F*_1,80_=0.88), body mass (*P*=0.10, *F*_1,80_=2.63; Fig. 3C) or body condition (*P*=0.91, *F*_1,80_=0.01).

Corticosterone treatment affected both SVL (Fig. S1) and body mass at hatching (*P*<0.001, *F*_2,123_=13.40; *P*=0.011, *F*_2,123_=8.93; Fig. 3A) but did not affect body condition (*P*=0.06, *F*_2,123_=5.43). Lizards treated with high doses of corticosterone had smaller SVLs than those treated with low doses of corticosterone (*P*=0.01) and control lizards (*P*=0.003). Lizards treated with high doses of corticosterone weighed less than control lizards (*P*=0.01) but did not differ in body mass compared with those treated with low doses of corticosterone (*P*=0.80). There were no differences in SVL or body mass measurements between lizards treated with low doses of corticosterone and control lizards (*P*=0.87 and 0.06). Corticosterone treatment during development affected juvenile body mass (*P*=0.03, *F*_2,101_=6.99; Fig. 3B) such that lizards exposed to high doses of corticosterone during development weighed less than control lizards (*P*=0.04) but were not different from lizards treated with low doses of corticosterone (*P*=0.12). There were no differences in body mass between lizards treated with low doses of corticosterone and control lizards (*P*=0.81). Corticosterone treatment during development did not affect juvenile SVL (*P*=0.20, *F*_2,101_=3.15) or body condition (*P*=0.71, *F*_2,101_=0.69). In adults, corticosterone treatment during development affected SVL (*P*=0.03, *F*_2,80_=6.86), with adults exposed to high doses of corticosterone during development having smaller SVLs as adults compared with control lizards (*P*=0.042) but not lizards with low doses of corticosterone (*P*=0.12). Additionally, there was no difference in adult SVL between lizards that received low doses of corticosterone during development and control lizards (*P*=0.95). Corticosterone treatment did not affect adult mass (*P*=0.20, *F*_2,80_=3.20; Fig. 3C) or body condition (*P*=0.34, *F*_1,80_=2.11).

Lizards incubated at warmer temperatures grew less in SVL compared with lizards incubated at cooler temperatures from hatching to the juvenile period (*P*<0.001, *F*_1,102_=12.83) and from hatching to adulthood (*P*<0.001, *F*_1,81_=11.69) but did not differ in growth of body mass from hatching to the juvenile period (*P*=0.26, *F*_1,102_=1.26) or hatching to adulthood (*P*=0.06, *F*_1,81_=3.52). Corticosterone treatment during development negatively affected growth of body mass from hatching to the juvenile period (*P*=0.042, *F*_2,102_=3.15). Lizards exposed to high doses of corticosterone grew more slowly than control lizards (*P*=0.046) but did not differ from low dose lizards (*P*=0.17). There was no difference in body mass gain between low dose and control lizards from hatching to the juvenile period (*P*=0.79). Corticosterone treatment during development did not affect the growth of SVL from hatching to the juvenile period (*P*=0.68, *F*_2,102_=0.76) or hatching to adulthood (*P*=0.57, *F*_2,81_=1.11) and did not affect change in body mass from hatching to adulthood (*P*=0.19, *F*_2,81_=3.28).

### Sustained effects of developmental treatments into adulthood – effects on hormones and mitochondrial bioenergetics

**Fig. 4.**
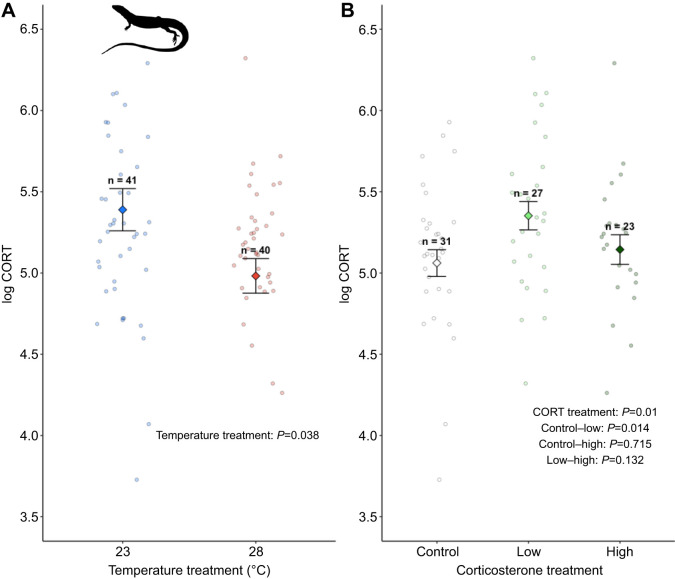
**Incubation temperature and corticosterone treatments during development affect baseline corticosterone levels in adults.** (A) Temperature treatment. (B) Corticosterone treatment. Data are from model results from emmeans, with mean and s.e. Sample sizes (*n*) for each treatment are indicated above error bars. Marginalised mean estimates (mean and s.e.) are provided based on a model that accounted for incubation temperature, hormone treatment, sex, age, test plate and clutch ID as a random factor.

Corticosterone treatment during development affected adult baseline corticosterone levels (*P*=0.045, *F*_2,68_=3.25; Fig. 4B). Lizards treated with low doses of corticosterone had higher levels of baseline corticosterone compared with control lizards but did not differ from lizards treated with high doses of corticosterone (control−low: *P*=0.01, low−high: *P*=0.13). There was no difference in baseline corticosterone levels between lizards treated with high doses of corticosterone and control lizards (*P*=0.72). Corticosterone treatment did not affect thyroxine (*P*=0.75, *F*_2,67_=0.56) or testosterone levels (Kruskal–Wallis chi-squared=0.19, *P*=0.91, *n*=12 control, *n*=16 low, *n*=12 high).

Incubation temperature affected baseline corticosterone levels in adult lizards (Fig. 4A; *P*=0.04, *F*_1,68_=4.29). Lizards incubated at a cooler temperature had higher baseline corticosterone compared with lizards incubated at a warmer temperature (log CORT cool 5.39±0.130, warm 4.98±0.106). In our experiment, lizards incubated at cooler temperatures were larger compared with lizards incubated at warmer temperatures (see above). Glucocorticoids are linked to energy demands in endotherms (e.g. Jimeno et al., 2020; Rubalcaba and Jimeno, 2022) and it is possible that cooler incubation temperatures indirectly affect baseline corticosterone levels through changes in body size. We conducted a *post hoc* analysis to test the potential effects of increased energy demands associated with larger body size on baseline corticosterone levels. When adult body mass was included as a covariate, we found that baseline corticosterone levels were positively correlated with adult body mass (*P*=0.01, *F*_1,67_=6.33) and did not differ between incubation treatments (*P*=0.21, *F*_1,65_=1.54). Incubation temperature treatment did not affect adult thyroxine (*P*=0.99, *F*_1,67_=0.001) or testosterone levels (Kruskal–Wallis chi-squared=0.06, *P*=0.81, *n*=21 cool, *n*=22 warm).

**Fig. 5.**
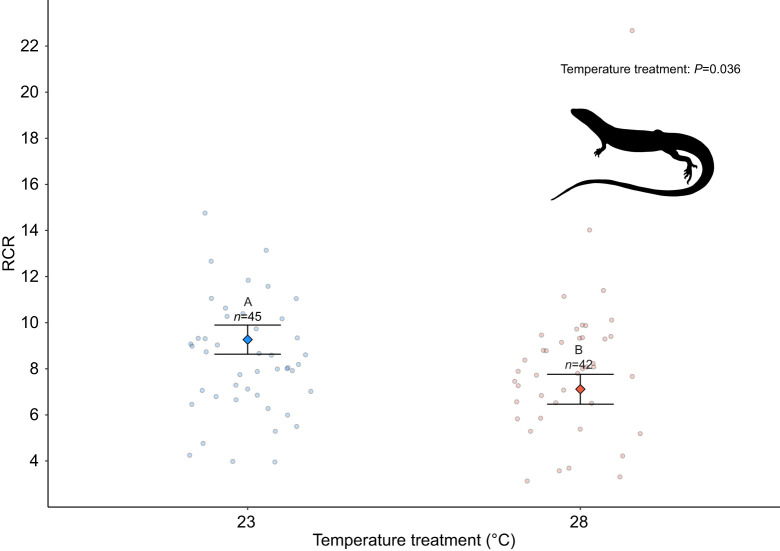
**Respiratory control ratio (RCR) in adults exposed to low or high incubation temperatures.** Significant differences (*P*<0.05) between effects of incubation temperature are indicated by different letters, and sample sizes (*n*) for each treatment are indicated above error bars. The model accounted for hormone, sex, body size and age. Clutch was treated as a random factor. Data are marginalised means and s.e. of developmental temperature used in the model.

**Fig. 6.**
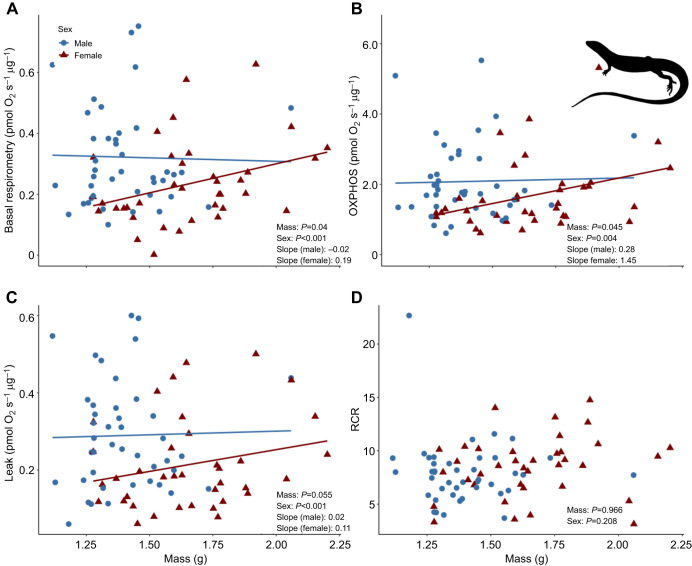
**Association between adult body mass and mitochondrial respiration parameters.** (A) Basal respiration, (B) OXPHOS respiration, (C) leak respiration and (D) RCR. Colours indicate sex and *P*-values in the bottom right of each panel indicate sex differences between treatments.

Mitochondrial efficiency (i.e. RCR) was affected by incubation temperature (*F*_1,77_=4.40, *P*=0.041; Table S2) but not corticosterone treatment (*F*_2,77_=2.14, *P*=0.34; Table S2). Lizards incubated at cooler temperatures had greater mitochondrial efficiency as indicated by higher RCRs than lizards incubated at warmer temperatures (Fig. 5). Developmental treatments did not affect basal, OXPHOS or leak respiration (Table S2). Basal and OXPHOS respiration were positively associated with adult body mass (*P*=0.04, 0.045; *F*_1,75_=4.21, 4.02; Fig. 6A,B) and leak respiration showed a near-significant relationship with body mass (*P*=0.055, *F*_1,75_=3.69; Fig. 6C). There was no association between RCR and body mass (*P*=0.97, *F*_1,75_=0.01; Fig. 6D).

Overall, females had higher baseline corticosterone levels (*P*=0.003, *F*_1,68_=8.5) and higher thyroxine levels than males (*P*=0.02, *F*_1,67_=5.36). Males had higher oxygen consumption than females for basal (*P*<0.001, *F*_1,75_=15.35), OXPHOS (*P*=0.004, *F*_1,75_=8.14) and leak respiration (*P*<0.001, *F*_1,75_=14.60). However, there was no difference between males and females in RCR values (*P*=0.21, *F*_1,75_=1.58).

### Associations between growth, mitochondrial bioenergetics and hormone levels

There were no associations between growth in body mass or SVL and mitochondrial respiration parameters from hatching to the juvenile period or hatching to adulthood (Tables S3 and S4). Growth in body mass from hatching to adulthood was positively associated with corticosterone levels for all models with mitochondrial respiration parameters, but these effects were not significant (basal: *F*_1,67_=3.36, *P*=0.07; OXPHOS: *F*_1,67_=3.06, *P*=0.08; leak: *F*_1,67_=3.56, *P*=0.06; RCR: *F*_1,67_=3.52, *P*=0.06). There were no associations between growth in SVL from hatching to adulthood and corticosterone levels for all models (basal: *F*_1,67_=1.41, *P*=0.23; OXPHOS: *F*_1,67_=1.08, *P*=0.30; leak: *F*_1,67_=1.66, *P*=0.20; RCR: *F*_1,67_=1.57, *P*=0.21). Body mass and SVL growth were lower for males than for females in all models (Tables S3 and S4). There were no associations between growth in body mass or SVL and thyroid hormone levels (Tables S3 and S4).

### Developmental temperature and corticosterone effects on mortality

Incubation temperature did not affect mortality across the duration of our study (cool temperature: *n*=6 of 56 deceased; warm temperature: *n*=9 of 54 deceased; chi-squared=0.27, d.f.=1, *P*=0.60). Similarly, corticosterone treatment did not affect mortality (high corticosterone: *n*=5 of 33 deceased; low corticosterone: *n*=5 of 38 deceased; control: *n*=5 of 39 deceased; chi-squared=0.07, d.f.=2, *P*=0.97).

## DISCUSSION

Developing animals can be affected by elevated temperatures associated with climate change directly through interactions with their environment and indirectly through maternal effects such as increased exposure to maternally derived glucocorticoids (Crino et al., 2024; Mentesana and Hau, 2022). Developing animals may be particularly affected by elevated temperatures associated with climate change because developmental conditions can have long-term effects on physiological traits (e.g. Cossin-Sevrin et al., 2022; Crino et al., 2022; Stier et al., 2022). We found evidence that incubation temperatures and exposure to prenatal corticosterone affect body size and physiological traits (baseline corticosterone and mitochondrial function) across lifespan. Contrary to our predictions, we found no evidence for interactive effects between incubation temperature and prenatal corticosterone exposure. Our results are consistent with past studies showing that incubation temperature can have sustained effects on physiological traits in oviparous vertebrates. Our results are the first to show that prenatal conditions can cause sustained changes to HPA axis function in lizards.

### Early thermal environment and corticosterone do not have interactive effects but independently affect physiology and growth

We found that incubation temperature and corticosterone treatment had independent effects on body size. Lizards incubated at warm incubation temperatures (28°C) or exposed to high levels of corticosterone prenatally were smaller compared to lizards incubated at cooler temperatures (23°C) and lizards not exposed to corticosterone (control). These treatment effects on body size were present at hatching and the juvenile period (temperature and corticosterone) and in adults (corticosterone treatment only). In oviparous lizards, elevated incubation temperatures generally decrease incubation duration and can result in smaller hatchlings in some species (Booth, 2018; Noble et al., 2018). The effects of incubation temperature on growth in oviparous lizards are likely due to the effects of temperature on energy metabolism (see discussion on mitochondrial function, below; Angilletta et al., 2002; Dowd et al., 2015; Salin et al., 2016). In our experiment, elevated temperature had sustained but not lifelong effects on body size, suggesting that lizards compensate for early developmental effects with changes in postnatal growth. Such compensatory growth has been linked to elevated production of reactive oxygen species (ROS), oxidative damage and faster senescence (Metcalfe and Monaghan, 2001; Monaghan et al., 2009). Compensatory growth has also been linked to elevated production of antioxidants that can mitigate the damaging effects of ROS (De Block and Stoks, 2008; Noguera et al., 2015). We found no effect of elevated incubation temperature on mortality across the duration of our study but did not measure ROS or antioxidant production. Future studies that assess the effects of incubation temperature and compensatory growth on ROS and antioxidant production could uncover mechanisms that shape long-term effects of developmental conditions.

We predicted that incubation temperature would affect the metabolism of yolk corticosterone and result in interactive effects between incubation temperature and corticosterone treatment because elevated incubation temperatures can increase embryonic metabolism (e.g. Angilletta et al., 2006; Booth et al., 2000). However, we did not see interactive effects between incubation temperature and corticosterone treatment that would support this prediction. It is possible that incubation temperature and corticosterone treatment did not have interactive effects because they affect developing embryos through different physiological pathways and/or across different time scales. In viviparous lizards, placental tissue metabolises corticosterone, which potentially buffers developing embryos from elevated levels of maternal corticosterone (Painter and Moore, 2005). Much less is known about how maternal corticosterone affects developing embryos in oviparous lizards. However, in birds, maternal corticosterone is metabolised into 5β-corticosterone and 20β-corticosterone in the extraembryonic membrane early in development (Vassallo et al., 2019, 2014). For example, studies showed that embryonic chickens (*Gallus gallus*) and Japanese quail (*Cortunix japonica*) metabolised ∼100% of yolk corticosterone during the first 4–6 days of development (Harders et al., 2024; Vassallo et al., 2014). To our knowledge, no study to date has characterised the rate of corticosterone metabolism by embryonic lizards. However, in red-eared slider turtles (*Trachemys scripta*), embryos metabolised ∼50% of topically applied oestradiol during the first 9 days of development (Crews et al., 1991). Together, these studies suggest that lizard embryos in our experiment were exposed to elevated corticosterone levels for a short period following treatment whereas temperature treatments affected lizards until hatching.

### Incubation temperature and prenatal corticosterone affect baseline corticosterone as adults

Exposure to elevated levels of glucocorticoids during development can affect HPA axis function across lifespan (reviewed in Gans and Coffman, 2021; Matthews and McGowan, 2019; Seckl and Meaney, 2004). We showed that prenatal exposure to corticosterone affects baseline corticosterone in adult lizards (average age 466.1 days post-hatching). Lizards exposed to low doses of corticosterone prenatally had higher levels of baseline corticosterone as adults compared to control lizards (Fig. 4B). However, we found no differences in corticosterone levels between lizards treated with high doses of corticosterone and lizards treated with low doses of corticosterone or the control treatment. The effects of glucocorticoid exposure during development on HPA axis function later in life are complex and vary by the dose or magnitude of glucocorticoid exposure, the longevity of effects, species, sex and postnatal conditions (Chaby, 2016; Gans and Coffman, 2021; Majer et al., 2023; Monaghan and Haussmann, 2015). For example, glucocorticoids can have biphasic dose-dependent effects where low doses elicit one response that is reversed at higher doses (resulting in an inverted U-shaped response curve; Gopi and Rattan, 2019; Lupien et al., 2005; Pratsinis et al., 2006). Such dose-dependent effects of glucocorticoids on HPA axis function could increase survival by priming glucocorticoid responses to match developing animals to their postnatal environment (a process called hormesis; Costantini et al., 2010; Monaghan and Haussmann, 2015). The duration of such glucocorticoid-mediated changes in HPA axis function has important implications for understanding the power of these developmental effects to shape evolutionary responses. For example, exposure to developmental glucocorticoids in one generation can affect future generations through intergenerational effects when glucocorticoid exposure during development has sustained effects on HPA axis function that persist until sexual maturity (Crino et al., 2024).

Environmental temperatures experienced during development can influence thermoregulation and temperature tolerance at later life-history stages (Esquerré et al., 2014; Goodman and Walguarnery, 2007; Nord and Giroud, 2020). Such developmental effects can be mediated through changes in the HPA axis and the thyroid hormone axis (hypothalamic–pituitary–thyroid axis), which plays an important role in behavioural and physiological thermoregulation (Debonne et al., 2008; Loyau et al., 2015; Wilsterman et al., 2015). Consistent with this idea, we found that lizards incubated at cooler temperatures had higher levels of baseline corticosterone compared with lizards incubated at warmer temperatures (Fig. 4A). Glucocorticoids regulate physiological processes that increase circulating levels of glucose and lipids by increasing hepatic glucose production (through gluconeogenesis) and by reducing glucose uptake by skeletal muscles (Picard et al., 2014; Sapolsky et al., 2000). As such, glucocorticoids are often linked to metabolic demands (Astheimer et al., 1992; Jimeno et al., 2018; Remage-Healey and Romero, 2000). In our experiment, lizards incubated at cooler temperatures were larger than lizards incubated at warmer temperatures at hatching and during the juvenile period (Fig. 3; Fig. S1). We found that larger lizards had higher levels of baseline corticosterone when body mass was included in a *post hoc* analysis (and no effect of incubation treatment). We also found no difference in thyroxine levels in adult lizards incubated at warm and cool temperatures. Together, these results suggest that incubation temperature does not have programmatic effects on endocrine mechanisms. Rather, incubation temperature drives sustained effects on body size and, hence, metabolic demands as reflected by differences in corticosterone levels in our experiment.

### Mitochondrial bioenergetics are affected by incubation temperature and differ between sexes

We found that incubation temperature had sustained effects on the efficiency of mitochondrial respiration (i.e. RCR; Fig. 5). The mitochondrial RCR is calculated as a ratio of oxygen consumed during State 3 respiration (OXPHOS; when ATP is synthesised) to oxygen consumed during State 4 respiration (leak; when protons flux across the inner mitochondrial membrane into the matrix without producing ATP; Gnaiger, 2012). Mitochondrial respiratory control is considered one of the best metrics of mitochondrial function in isolated mitochondria because it is affected by numerous biochemical factors and captures a biologically relevant metric of mitochondrial efficiency (Brand and Nicholls, 2011). High mitochondrial RCRs indicate that mitochondria have a high capacity for substrate oxidation and ATP turnover relative to a low loss of potential energy (as heat) due to proton leak (Brand and Nicholls, 2011).

In our experiment, lizards incubated at cooler temperatures had higher mitochondrial efficiency (higher RCR) as adults compared with lizards incubated at warmer temperatures. The liver plays a central role in glucose and lipid metabolism (Han et al., 2016; Jones, 2016). As such, higher efficiency mitochondria may explain why we observed larger body size in lizards incubated at cooler temperatures because more ATP is expected be available early in development for growth and somatic maintenance. Our results also suggest that temperatures experienced during early development can have sustained effects on the efficiency of hepatic mitochondria, possibly through changes in membrane fluidity and/or lipid profile differences that can affect the ‘leakiness’ of mitochondrial membranes to protons. Membrane fluidity is affected by membrane phospholipid characteristics, varies in response to temperature, and is considered an important mechanism that promotes thermal adaptation in ectotherms (i.e. homeoviscous adaptation; Chung and Schulte, 2020; Cooper et al., 2014; Dahlhoff and Somero, 1993). Work in *Drosophila* has shown that the ability to plastically change phospholipid composition in response to thermal environments varies across populations (Cooper et al., 2014). Exploring how lipid membranes change in response to incubation temperature (if at all) would be a fruitful future endeavour to test a potential mechanism through which developmental conditions affect mitochondrial efficiency.

Independent of developmental treatments, in our experiment males had higher mitochondrial oxygen consumption than females for basal, OXPHOS and leak respiration (Fig. 6). Oxygen consumed during OXPHOS respiration is used to drive the phosphorylation of ADP to ATP while leak respiration is a measure of oxygen consumption used to offset proton loss across the inner mitochondrial membrane and is reflective of energy loss (Brand and Nicholls, 2011). Our results suggest that males have a greater ability to produce ATP but also require more energy to offset proton leak than females. However, we found no difference in RCR values between males and females, indicating no difference between the capacity for energy production relative to energy loss, suggesting that males have overall higher mitochondrial function compared to females despite their smaller body size and slower growth rate. An enhanced ability of mitochondria to produce ATP may account for variation in metabolically costly processes and traits other than growth, such as thermal tolerance, reproduction, and sexual displays and ornaments (Chung and Schulte, 2020; Hill, 2014; Koch and Hill, 2018).

### Conclusion

Understanding the short-term and sustained effects of developmental conditions on growth, body condition and survival is essential for understanding how developmental effects drive population-level responses. However, the interaction between physiological systems and environmental conditions likely entails physiological trade-offs that constrain phenotypic expression and ultimately affect life-history strategies. For this reason, it is critical to understand how developmental conditions interact during sensitive periods such as prenatal development. Oviparous animals could be affected by elevated temperatures associated with global climate change through direct effects on incubation temperature and maternal effects such as increased exposure to glucocorticoids. Studies that track the physiological changes to elevated temperatures and glucocorticoids during development across lifespan will provide a more holistic understanding of the multigenerational consequences of elevated temperatures associated with global climate change.

## Supplementary Material

10.1242/jexbio.249234_sup1 Supplementary information
