# Supplementary material for: From eggs to adulthood: sustained effects of early developmental temperature and corticosterone exposure on physiology and body size in an Australian lizard
Source: J Exp Biol. 2024 Dec 12;227(24):jeb249234. doi: 10.1242/jeb.249234 (PMC11655029; doi:10.1242/jeb.249234)
Supplement: Supplementary information [file jexbio-227-249234-s1.pdf]

Supplementary Materials and Methods

Measurements of yolk corticosterone levels

To remove the egg yolk, we made a lateral incision with a razor blade on the eggshell. We used dissection scissors to widen the incision and removed the egg yolk using a small spatula. We used KimWipes to remove any albumin that surrounded the egg yolk. Egg yolks were weighed to the nearest in 0.001 mg using a Sartorius microbalance, diluted with 1 mL of doubly distilled water, vortexed thoroughly, and stored at -20º C until assayed.

We used solid phase extraction (SPE) with silica bonded vacuum columns (United Chem. Cat. No. CEC18156) to extract corticosterone from yolk samples. We prepared columns by washing them twice with 5 mL of doubly deionized water. Diluted yolk samples were run through the columns, followed by a wash with 5 mL of 40% methanol to remove lipids. Columns were then soaked with 5 mL of 100% methanol for 2 minutes before samples were eluted with vacuum filtration. Samples were dried under nitrogen at 35º C and then stored at -20º C until assayed. To determine extraction efficiency, we pooled yolk samples and used SPE to extract corticosterone from an aliquot that was spiked with 1,000 pg of corticosterone and an aliquot that was not spiked. We calculated one value of extraction efficiency for each group of samples that was assayed on a hormone plate with an average extraction efficiency across all plates of 25.2%.

We measured yolk corticosterone levels using Arbor Assay Enzyme Immunoassay (EIA) kits (Cat. No. K014). Following extraction, samples were reconstituted in 600 µL of assay buffer, vortexed thoroughly, and mixed on a test tube shaker for 20 minutes. An external standard of 500 pg/mL was run on every plate and used to calculate inter-plate variation. All samples and standards were run in triplicate. Plates were read on FLUOstar Omega microplate readers at 450 nm. Corticosterone levels were calculated from a four parameter nine-point standard curve ranging from 39.063 to 10,000 pg/mL. Intra- and inter- plate variation was 2.05 and 6.05% respectively. We tested assay parallelism by serially diluting a pooled yolk extract and comparing the slope of the antibody binding to that of the standard supplied with the EIA kits. The serial dilution curve was parallel to the standard curve ($p = 0.22$, $F_{1,7} = 1.83$).

Determining protein concentration of mitochondrial suspensions

We used Coomassie Plus (Bradford) assays (Thermo Scientific, Cat. No. 23236) to determine the protein concentration of liver mitochondria suspensions. Sample concentration

Journal of Experimental Biology • Supplementary information

was determined from 400 µL aliquots of mitochondrial suspension. We separated mitochondria from the respiration media prior to assays. Briefly, we centrifuged aliquots at 4ºC, 10,000 x g for 10 minutes. We removed and discarded the supernatant and resuspended the pelleted mitochondria in 400 µL of doubly distilled water. We centrifuged the re-suspended aliquots at 4ºC, 10,000 x g for 10 minutes. We removed and discarded the supernatant and dried the pelleted mitochondria under nitrogen (~20 minutes). We re-suspended the dried pellet in 100 µL of doubly distilled water. Samples were assayed in triplicate from 20 µl aliquots of the washed mitochondrial suspension. A pooled sample of washed mitochondrial suspension was run on every plate and used to calculate inter-plate variation. Plates were read on a FLUOstar Omega microplate reader at 595 nm. Protein values were determined from a 10-point standard curve (7.8125 – 1000 ug/mL) and corrected by the dilution factor. Intra- and inter-plate variation for was 3.76 and 8.99% respectively.

Plasma hormone levels

Immediately following euthanasia and decapitation, trunk blood was collected using heparinized microcapillary tubes and kept on ice until processing (>1 hour). Blood was centrifuged at 7,000 rpm for 7 minutes and the isolated plasma was removed and stored at - 20ºC until assayed. We were unable to collect 5 µl of plasma from n = 7 individuals and instead assayed samples of 2 – 4 µl of plasma. These samples were diluted to [1:100] and assayed as per samples of 5µl of plasma. Prior to reading the plate, we were able to distinguish the samples assayed from small volumes due to color differences, suggesting that there was sampling error associated with small volumes. Although these samples were assayed, they were excluded prior to statistical analyses to avoid error associated with small volumes.

Plasma corticosterone levels were quantified using methods described above for measuring yolk corticosterone levels. Intra- and inter-plate variation for plasma corticosterone assays was 2.25 and 3.63% respectively. Thyroxine (T4) levels were quantified with Arbor Assay kits (Cat. No. K050). An external standard of 1,000 ng/mL was run on every plate and used to calculate inter-plate variation. Thyroxine levels were calculated from a 4 parameter six-point standard curve ranging from 0.625 to 20 ng/mL. Intra- and inter-plate variation for thyroxine assays was 3.91 and 7.0% respectively. Testosterone levels in males were quantified with Arbor Assay kits (Cat. No. K032). An external standard of 400 pg/ml was run on every plate and used to calculate inter-plate variation. Testosterone levels were calculated from a linear six-point standard curve ranging from 40.96 to 10,000 pg/mL. A

Journal of Experimental Biology • Supplementary information

linear fit was used for the standard curve to calculate high values that were not captured with a 4-parameter fit. We used rank order statistics to analyze testosterone data (see below) because linear standard curves may underestimate high hormone values. Intra- and inter-plate variation for testosterone assays was 1.30 and 3.35% respectively.

Statistical analysis

We used a general linear effects model (GLM) to test differences in yolk corticosterone levels following treatment with log transformed corticosterone levels as the dependent variable and corticosterone treatment and assay plate ID as fixed factors. Sample sizes were n = 10 for high corticosterone, n = 8 for low corticosterone, and n = 8 for control. One yolk treated with the high corticosterone treatment had yolk a corticosterone level over two standard deviations greater than mean corticosterone levels. We conducted separate GLMs with and without this datum. The results were statistically equivalent, and it was included in the final model.

We used generalized linear mixed effects models (GLMMs) using the *lme4* package (1.1.35.3), in R version 4.4.0. In all models, we included clutch of origin as a random effect to account for lizards originating from the same mother. To test the effects of developmental treatments on body size and condition we used SVL, body mass, or body condition as the dependent variables (all assumed to follow a Gaussian error distribution with identity link function) and temperature treatment and corticosterone treatment as fixed factors. For models that tested treatment effects post-hatching, we also included a covariate of the days post- hatching to account for the variation in the ages of the lizards. We included sex as a fixed factor in models that tested the effects of treatments on adult body size measurements. We tested treatment effects on growth using growth scores for SVL and mass measurements as dependent variables and temperature and corticosterone treatment and sex (adults only) as fixed factors.

We also used GLMMs to test the effects of developmental treatments on adult corticosterone and thyroxine levels. We first fit GLMMs to test the effects of the time to collect blood samples and the assay plate ID (to account for inter-assay variation) on log transformed hormone levels. The time to collect blood samples did not affect corticosterone or thyroxine levels ($p = 0.77$, $F_{1,75} = 0.09$; $p=0.29$, $F_{1,71} = 1.12$, respectively), but did positively affect testosterone levels ($p = 0.04$, $F_{1,40} = 4.21$). Plate ID affected corticosterone levels ($p = 0.007$, $F_{4,75} = 19.6$) but not thyroxine or testosterone levels ($p = 0.29$, $F_{3,71} = 1.12$;

Journal of Experimental Biology • Supplementary information

$p = 0.22$, $F_{1,40} = 1.49$). Significant factors were included in models to test for treatment effects on hormone levels, but if were not significant were removed from models to ease their interpretation. We used log transformed corticosterone or thyroxine levels as dependent factors, temperature and incubation treatments and sex as fixed factors, and scaled age as a covariate. Again, we used a Gaussian error distribution with an identity link function. We used residuals of testosterone level regressed against blood collection time to test effects of developmental treatments on testosterone levels in males using Kruskal-Wallis tests.

To test the effects of developmental treatments on mitochondrial bioenergetics, we created GLMMs with each respiration state (basal, OXPHOS, and leak) and the RCR as the dependent variables and incubation temperature, corticosterone treatment, sex, and respirometer chamber nested in respirometer identity as fixed factors and scaled age as a covariate. We compared models with and without respirometry chamber to determine if variance in each dependent variable was driven by chamber variation. In all cases, it was not important, so we removed this factor from models to ease interpretation. We tested associations between mitochondrial bioenergetics and endogenous corticosterone, thyroxine, and testosterone (males only) levels using GLMMs with respiration state and RCR as dependent variables, log transformed corticosterone and thyroxine levels as covariates, and sex and respirometer chamber nested in respirometer identity as fixed factors.

We tested associations between growth and physiological parameters using GLMMs with growth of mass or SVL as dependent variables, thyroxine levels, corticosterone levels, and mitochondrial respiration parameters as covariates, and sex as a fixed factor. We used log transformed corticosterone and thyroxine levels in GLMMs. We did not account for handling time to collect blood samples or plate identity in this set of GLMs because these factors did not affect hormone levels in this subset of data. We constructed separate GLMMs for each mitochondrial respiration state because basal, OXPHOS, and leak respiration were highly correlated ($p < 0.001$, $r > 0.81$ for all).

Journal of Experimental Biology • Supplementary information

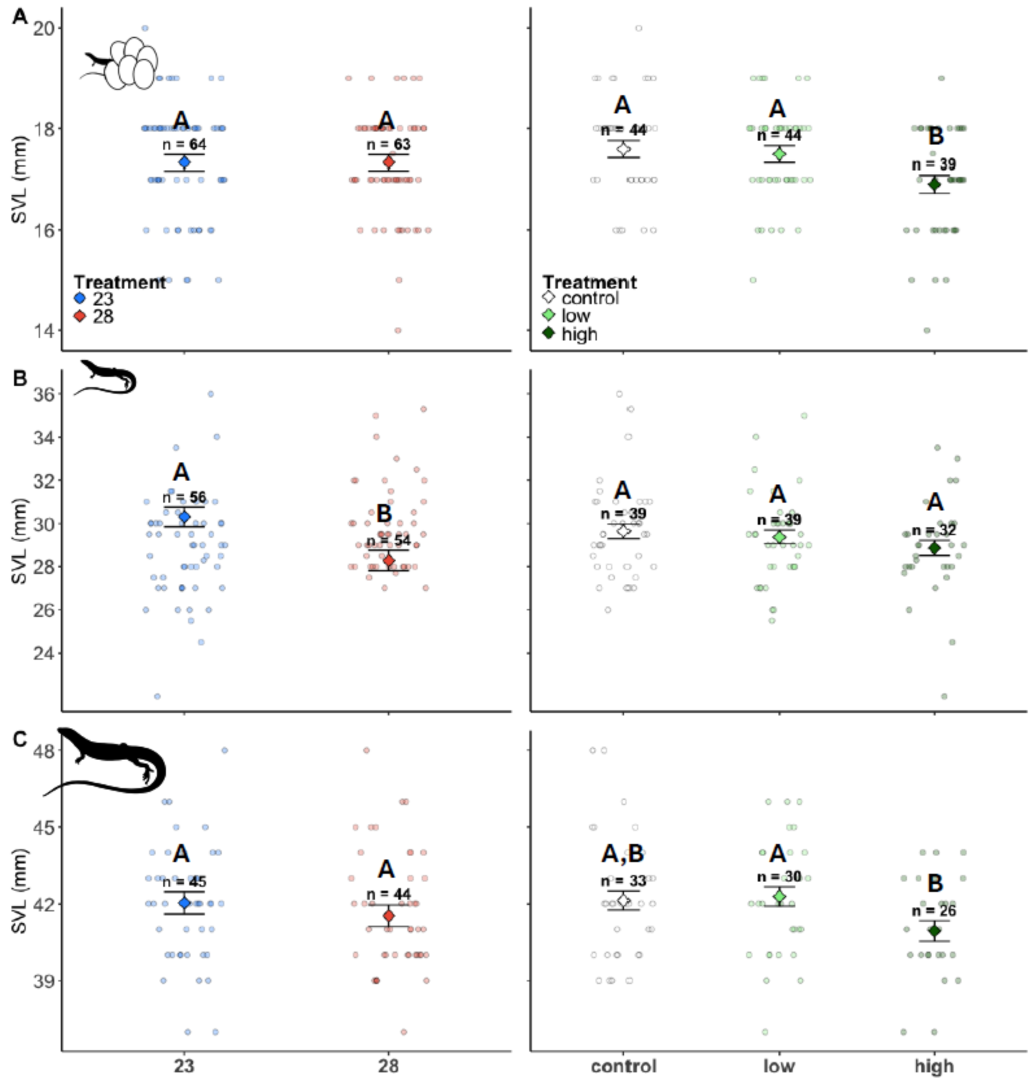

Fig. S1. Snout vent length (SVL) at hatching (A), the juvenile period (B), and adulthood (C) in lizards exposed to incubation treatments (left panels) and prenatal corticosterone treatments (right panels). Significant differences ($p < 0.05$) from main effects of incubation temperature and post hoc tests for differences between corticosterone treatments are indicated by different letters and sample sizes (n) for each treatment are indicated above. Marginalized mean estimates (mean and SE) are provided based on a model that accounted for incubation temperature, hormone treatment, body size, age, and clutch ID as a random factor.

Journal of Experimental Biology • Supplementary information

Table S1. Model comparisons between main effects (temperature + hormone) and interaction model (temperature * hormone interaction) using AIC for the different response variables measured. Note that the AIC of models containing the interaction are subtracted from the AIC of models containing just the main effects. Positive values indicate main effects models had lower AIC and were therefore better supported. If models were within 2 AIC units of each other we simplified to the model with fewer parameters (i.e., main effects model). All models were fit using maximum likelihood for model selection. For details on the specific models fit for each response variable see the Statistical Analysis section.

Variable	Delta_AIC
Incubation time (days)	3.83
Hatchling Snout-vent Length (SVL) (mm)	3.58
Hatchling Mass (g)	3.47
Juvenile SVL (mm)	1.75
Adult SVL (mm)	0.76
Juvenile Mass (g)	3.01
Adult Mass (g)	2.58
Hatchling to Juvenile Growth SVL (mm)	2.25
Hatchling to Juvenile Growth Mass (g)	3.08
Hatchling to Adult Growth SVL (mm)	0.37
Hatchling to Adult Growth Mass (g)	2.97
Basal respiration (pmol/sec/ng)	3.77
OXPHOS respiration (pmol/sec/ng)	3.97
LEAK respiration (pmol/sec/ng)	3.22
Respiratory Control Ratio (RCR)	1.95

Journal of Experimental Biology • Supplementary information

Table S2. Summary of GLMs testing the effects of developmental treatments on mitochondrial respiration (basal, OXPHOS, leak, RCR). Significant terms (p<0.05) are highlighted in bold.

Test	term	statistic	df	p.value
Basal	hormone	2.929	2	0.231
	scale(adult_age)	1.262	1	0.261
	**scale(adult_mass_g)**	**4.213**	**1**	**0.040**
	**sex**	**15.358**	**1**	**0.000**
	temp	0.232	1	0.630
LEAK	hormone	2.185	2	0.335
	scale(adult_age)	2.522	1	0.112
	scale(adult_mass_g)	3.691	1	0.055
	**sex**	**14.608**	**1**	**0.000**
	temp	1.496	1	0.221
OXPHOS	hormone	3.917	2	0.141
	scale(adult_age)	0.086	1	0.769
	**scale(adult_mass_g)**	**4.025**	**1**	**0.045**
	**sex**	**8.140**	**1**	**0.004**
	temp	1.291	1	0.256
RCR	hormone	2.139	2	0.343
	scale(adult_age)	3.730	1	0.053
	scale(adult_mass_g)	0.002	1	0.966
	sex	1.585	1	0.208
Test	term	statistic	df	p.value
	**temp**	**4.399**	**1**	**0.036**

Journal of Experimental Biology • Supplementary information

Table S3. Summary of GLMs testing the effects of mitochondrial respiration (basal, OXPHOS, leak, and RCR), sex, baseline corticosterone levels, and thyroxine levels on growth rate (change in mass). Corticosterone and thyroxine levels were log transformed prior to analyses. Significant terms ($p<0.05$) are highlighted in bold.

Test	term	statistic	df	p.value
Growth mass (mg/d)	basal_corrected_pmol	1.104	1	0.293
	log(CORT_Final_Hormone_ng_mL)	1.410	1	0.235
	log(T4_corrected_ng_mL)	0.093	1	0.761
	**sex**	**8.288**	**1**	**0.004**
Growth mass (mg/d)	log(CORT_Final_Hormone_ng_mL)	1.662	1	0.197
	log(T4_corrected_ng_mL)	0.074	1	0.785
	oligo_corrected_pmol	0.981	1	0.322
	**sex**	**8.196**	**1**	**0.004**
Growth mass (mg/d)	adp_corrected_pmol	2.761	1	0.097
	log(CORT_Final_Hormone_ng_mL)	1.088	1	0.297
	log(T4_corrected_ng_mL)	0.023	1	0.880
	**sex**	**9.351**	**1**	**0.002**
Growth mass (mg/d)	RCR	0.338	1	0.561
	log(CORT_Final_Hormone_ng_mL)	1.567	1	0.211
	log(T4_corrected_ng_mL)	0.026	1	0.871
	**sex**	**6.745**	**1**	**0.009**

Journal of Experimental Biology • Supplementary information

Table S4. Summary of GLMs testing the effects of mitochondrial respiration (basal, OXPHOS, leak, and RCR) corrected for protein content, sex, baseline corticosterone levels, and thyroxine levels on growth rate (change in SVL). Corticosterone and thyroxine levels were log transformed prior to analyses. Significant terms are highlighted in bold.

Test	term	statistic	df	p.value
Growth SVL (mm/d)	log(CORT_Final_Hormone_ng_mL)	0.081	1	0.776
	log(T4_corrected_ng_mL)	0.195	1	0.659
	**sex**	**11.073**	**1**	**0.001**
Growth SVL (mm/d)	log(CORT_Final_Hormone_ng_mL)	0.286	1	0.593
	log(T4_corrected_ng_mL)	0.009	1	0.923
	**sex**	**12.797**	**1**	**0.000**
Growth SVL (mm/d)	log(CORT_Final_Hormone_ng_mL)	0.256	1	0.613
	log(T4_corrected_ng_mL)	0.425	1	0.515
	**sex**	**9.526**	**1**	**0.002**
Growth SVL (mm/d)	log(CORT_Final_Hormone_ng_mL)	1.008	1	0.315
	log(T4_corrected_ng_mL)	2.039	1	0.153
	sex	0.379	1	0.538

Journal of Experimental Biology • Supplementary information
